# Cryptanalyzing and Improving an Image Encryption Algorithm Based on Chaotic Dual Scrambling of Pixel Position and Bit

**DOI:** 10.3390/e25030400

**Published:** 2023-02-22

**Authors:** Shuqin Zhu, Congxu Zhu, Hanyu Yan

**Affiliations:** 1 School of Computer Science and Technology, Liaocheng University, Liaocheng 252059, China; 2 School of Computer Science and Engineering, Central South University, Changsha 410083, China

**Keywords:** cryptanalysis, chosen plaintext attack, image encryption, chaotic system

## Abstract

An image encryption algorithm for the double scrambling of the pixel position and bit was cryptanalyzed. In the original image encryption algorithm, the positions of pixels were shuffled totally with the chaotic sequence. Then, the 0 and 1-bit positions of image pixels were scrambled through the use of another chaotic sequence generated by the input key. The authors claimed that the algorithm was able to resist the chosen-plaintext attack. However, through the analysis of the encryption algorithm, it was found that the equivalent key of the whole encryption algorithm was the scrambling sequence *T* in the global scrambling stage, the pixel bit level scrambling sequence *WT* and the diffusion sequence *S*. The generation of scrambling sequence *T* is related to the sum of all pixel values of the plaintext image, while the generation of *WT* and *S* is not associated with the image to be encrypted. By using a chosen-plaintext attack, these equivalent key streams can be cracked so as to realize the decoding of the original chaotic encryption algorithm. Both theoretical analysis and experimental results verify the feasibility of the chosen-plaintext attack strategy. Finally, an improved algorithm was proposed to overcome the defect, which can resist the chosen-plaintext attack and has the encryption effect of a “one time pad”.

## 1. Introduction

As we all know, all kinds of information are transmitted and stored through the network. Digital images have been widely used as a data format. Because digital images contain some personal privacy information or important confidential information, how to protect images is an important topic. For the security of image information, scholars have proposed many technologies, such as data hiding [1], encryption [2], and watermarking [3]. Among these technologies, image encryption technology is the most direct method, which converts the original visual image to a noise image [4,5]. Generally speaking, image encryption algorithms include scrambling and diffusion algorithms. The scrambling algorithm means changing the pixel position, while the diffusion algorithm means changing the pixel value. Due to the particularity of chaotic sequences, such as pseudo-random, aperiodic, and highly sensitive to control parameters and initial conditions, and the ability to generate a large number of chaotic sequences quickly and accurately, these characteristics make chaotic systems especially suitable for image encryption. Since Fridrich [6] first designed an image encryption algorithm based on a 2D chaotic map, researchers have designed various image encryption algorithms using chaotic systems [7,8,9,10,11,12,13,14]. Here, some typical image encryption algorithms are introduced as follows. Yang et al. [7] analyzed the dynamic characteristics of the fractional order laser chaotic system and designed an image encryption algorithm. In order to overcome the difficulty of key management in a “one-time pad” encryption scheme and resist the attack of chosen plaintext, Zhu et al. [8] proposed an image encryption algorithm based on chaos and SHA-256. The algorithm adopted the ciphertext feedback mechanism of a dynamic index; that is, the position index of the ciphertext used for feedback was dynamic and could ensure that the algorithm resisted the attack of chosen plaintext and overcame the difficulty of key management in a “one-time pad” encryption scheme. Xu et al. [9] proposed a chaotic image encryption algorithm based on block scrambling and dynamic index diffusion, which adopted traditional scrambling diffusion encryption architecture. In order to resist the chosen-plaintext attack, the dynamic indexing ciphertext feedback mechanism and the dynamic indexing plaintext feedback mechanism were adopted in the diffusion stage, which is also the main innovation of this paper. In Ref. [10], a new image encryption algorithm using the hyperchaotic system and Fibonacci Q-matrix was proposed, in which the original image was confused by utilizing randomly generated numbers through the six-dimension hyperchaotic system, and the permutated image was diffused using the Fibonacci Q-matrix. Zhu et al. [11] proposed a fast image encryption algorithm based on double chaotic S-boxes. In Ref. [12], a six-dimensional discrete chaotic system with a simple sine function was first constructed, and a chaotic pseudorandom number generator based on the system was designed. Then, an encryption algorithm with both a key avalanche effect and plaintext avalanche effect was proposed. The biggest feature of this algorithm is that it has an “avalanche effect”. In other words, due to the wrong key, the decrypted ciphertext becomes a white image with several “black spots” instead of a random chaotic image, which makes the encryption system more secure. Li et al. [13] proposed a multi-image encryption scheme based on the DNA-chaos algorithm. In this scheme, multiple images are merged into one image by a computational integral imaging algorithm, which significantly improves the efficiency of image encryption. A fast image encryption algorithm based on logistics-sine-cosine mapping was proposed in Ref. [14]. The algorithm first generates five sets of encrypted sequences from the logistics-sine-cosine mapping, then uses the order of the encryption sequence to scramble the image pixels and designs a new pixel diffusion network to further improve the key sensitivity and plain-image sensitivity of the encryption algorithm.

In addition, some algorithms also combine the idea of DNA coding [15,16,17]. For example, Rehman et al. [15] proposed an image encryption algorithm based on DNA encoding and mixed pixel replacement. The main feature of this algorithm is that it adopted the mixed pixel replacement diffusion method in the diffusion stage, which had the advantage of high encryption efficiency. In Ref. [16], a five-dimensional continuous hyperchaotic system was constructed, and an image encryption scheme based on the hyperchaotic system was proposed, which uses a DNA dynamic coding mechanism and a classical scrambling diffusion encryption structure. In the diffusion stage, the two-round diffusion method is used to dynamically change the DNA coding (DNA decoding) rules according to the pixel value of the plaintext image; that is, different images are encrypted with different DNA coding (DNA decoding) rules, which makes the algorithm resistant to the attack of chosen plaintext. On the other hand, a chaotic image encryption algorithm based on bit-level technology has attracted researchers’ attention due to its reliability and effectiveness. An image encryption algorithm based on bit position transformation and DNA coding was proposed in Ref. [17], in which a six-dimensional hyper-chaotic system was used, and the key stream generated by the hyper-chaotic system relates to the plaintext image.

At the same time, it is very important to analyze the image encryption algorithm based on chaos from the perspective of modern cryptography. In fact, cryptographic analysis and cryptographic design promote each other and are a contradictory unity. Cryptographic analysis can help crypto designers find out security vulnerabilities and improve the level of encryption algorithm design. Many image encryption algorithms based on chaos have been broken [18,19,20,21,22,23,24]. Li et al. [18] pointed out the following fact: for the image encryption algorithm with permutation structure, the critical task of cracking the secret key is to crack the permutation matrix, not the key itself. Because the permutation matrix is equivalent to the key, the image encryption algorithm with permutation structure can be cracked by a chosen plaintext attack. Chen et al. [19] proposed an efficient chosen-plaintext attack to a medical privacy protection scheme and disclosed its equivalent secret key by a (log_256_(3 × *M* × *N*) + 4) pair of chosen plain images and the corresponding cipher images, where *M* × *N* and ‘3′ are the size of the RGB color image and the number of color channels, respectively. The cryptanalysis of a hybrid secure image encryption scheme based on Julia set, and three dimensional Lorenz chaotic system was presented in [20], in which a practical chosen-plaintext attack was performed, which reveals that the cryptosystem, depending on only multiplication and bitwise XOR operation, could be effortlessly attacked. Ma et al. [21] gave a thorough security analysis of an image block encryption algorithm based on chaotic maps and found some critical security defects in the algorithm; then, the authors obtained an equivalent secret key from five chosen plain images and the corresponding cipher images. Zhu et al. [22] analyzed the security of image encryption systems based on bit-plane extraction and multi-chaos and recovered the equivalent diffusion key and the equivalent permutation key by only two special plaintext images and their corresponding cipher images. Liu et al. [23] analyzed the security performance of an image encryption algorithm based on a first-order time-delay system IEATD and the enhanced version IEACD and designed an efficient chosen-plaintext attack, and verified it with extensive experiments. Zhang [24] analyzed an image cryptosystem based on circular inter-intra pixels bit-level permutation and found some defects of it, such as its row-based scrambling being invalid for the special images and lacking diffusion operations in its processes. Meanwhile, the image encryption algorithm was cracked through the use of only a pair of chosen plain-cipher images or a pair of known plain-cipher images. It can be seen that the main reason for the above algorithms to be cracked is that the keystream used by the encryption system is independent of the image to be encrypted, which makes the encryption system unable to resist the chosen-plaintext attack and chosen-ciphertext attack.

Deng et al. [25] proposed an image chaotic encryption algorithm with the double scrambling of the pixel position and bit. The algorithm uses Kent chaotic mapping to generate the key sequence and generates the parameters of the chaotic system and the number of pre-iterations, respectively, according to the characteristics of the plaintext pixel value and the input key. First, chaotic sequences were used to realize the global scrambling of image pixel positions. Secondly, according to another newly generated chaotic sequence, the 0-bit and 1-bit image pixel values were scrambled. The algorithm has the advantages of simple encryption and large key space and can resist the attacks of statistical analysis and differential analysis. However, through our in-depth analysis, it was found that the algorithm could not resist the attack of chosen plaintext. On the assumption that the sum of all the pixels of the plaintext image was an input parameter, we could successively crack three equivalent key streams of the encryption algorithm by 9 + $ceil(\log_{256}m)+$ $ceil(\log_{256}n)$ plaintext gray images and their corresponding ciphertext images.

This paper is arranged as follows: In Section 2, we briefly introduce the original encryption algorithm in reference [25]. In Section 3, the security of the original encryption algorithms in the literature [25] is analyzed, and the security vulnerabilities are found. The equivalent key streams of the original algorithm can be cracked one by one by using the chosen plaintext attack method. In Section 4, the proposed attack algorithm is simulated. An improved algorithm is proposed based on the original algorithm that can resist the chosen-plaintext attack in Section 5. In Section 6, the security of the improved algorithm is simulated and analyzed, and its superiority in resisting attacks is verified. Finally, the summary and conclusion of the full text are given in Section 7.

## 2. Description of the Original Encryption Algorithm

The original encryption algorithm to be analyzed in this paper is from reference [25]. In this section, we first undertake a concise description of this algorithm.

### 2.1. Chaotic System

The chaotic system used in the original encryption system is the Kent map [26], and its mapping function is shown in Equation (1).

(1) $$x_{i+1}=F\left(x_{i}\right)=\left\{\begin{array}{c} \frac{x_{i}}{a},x_{i}\in (0,a]\\ \frac{1-x_{i}}{1-a},x_{i}\in (a,1) \end{array}\right.$$

**Figure 1:**
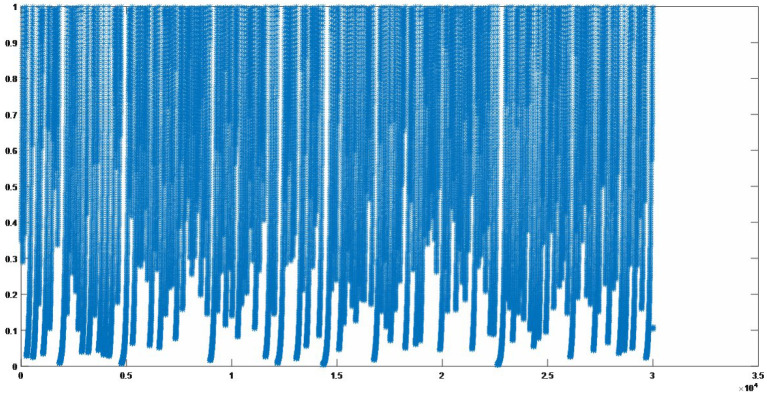
Iterative results of the chaotic map (1).

When the initial value *x*_0_ of the Kent map satisfies *x*_0_ ∈ (0, 1), and the control parameter *a* satisfies *a* ∈ (0, 1), the Kent map has a positive Lyapunov exponent, which indicates that the Kent map is a chaotic system. We take the initial value *x*_0_ = 0.43765, *a* = 0.8976; the results obtained by 30,000 iterations of Equation (1) are shown in Figure 1. It can be seen from Figure 1 that the iteration value covers the entire interval, which indicates that system (1) has chaotic characteristics such as pseudorandom, boundedness, ergodicity, etc.

### 2.2. Encryption Process

The encryption algorithm mainly includes two parts. The first part is the global scrambling based on pixel position. The second part is the scrambling process based on the bit level and the diffusion operation of the intermediate ciphertext.

#### 2.2.1. Global Scrambling of Pixel Positions

The pixel position scrambling operation is mainly to break the correlation of adjacent pixels. The specific steps are as follows:

**Step 1:** Convert the digital image matrix A with the size of *m* × *n* into a one-dimensional sequence *P* with the length of *m × n*. *P =* (*p*(1), *p*(2), …, *p*(*mn*))

**Step 2:** Calculate the sum of all the pixel values, and use Formulas (2) and (3) to calculate the control parameter *a* of the Kent mapping and pre-iteration number *K* + *mn* of the Kent map, respectively.

(2) $$a=sum/10^{8}$$

(3) K=1000+mod(sum,1000)

**Step 3:** Take the result *a* calculated by the formula (2) as the control parameter of the Kent map, set *x*_0_ as the initial value of Kthe ent map, and iterate the Kent map *K* + *mn* times to generate a chaotic sequence with the length of *K* + *mn*. Discard the first *K* values to obtain the sequence *L* with the length of *mn*. *L* = (*l*(1), *l*(2), …, *l*(*mn*)).The chaotic sequence *L* is sorted in ascending order to obtain the ordered sequence *L*’ = (*l*’(1), *l’*(2), …, *l’*(*mn*)) and the new sequence *T* = (*t*(1), *t*(2), …, *t*(*mn*)) is used to record the position of each element in the original sequence *L*

**Step 4:** Use the sequence *T* to scramble the plaintext sequence P according to the Formula (4) to obtain scrambled image *P*’ = (*p*’(1), *p’*(2), …, *p’*(*mn*))

(4) $$p^{\prime }\left(i\right)=p\left(t\left(i\right)\right),i=1,2,3,\ldots ,mn$$

It can be seen that the generation of the sequence *T* in the global scrambling stage is related to the image to be encrypted. Different encrypted images have different scrambling sequences *T*.

#### 2.2.2. Scrambling and Diffusion Based on the Bit Level of Pixel Values

**Step 1:** Reset the control parameter *a*_2_ of the Kent mapping and iterate Kent map *K*_2_ + *mn* times to generate a chaotic sequence with the length of *K*_2_ + *mn*. Discard the first *K*_2_ values to obtain the sequence *D* with the length of *mn*, where *D* = (*d*(1), *d*(2), …, *d*(*mn*)).

**Step 2:** For the *i*th real number *d* (*i*) in the chaotic sequence D, extracts eight digits after the decimal point of *d* (*i*) to form the sequence W = {W (1), W (2), W (3), …, W (8)}. For example, if the chaotic sequence value *d* (*i*) = 0.568 972 344, the generated sequence is W = {5, 6, 8, 9, 7, 2, 3, 4}. Sort the sequence W in ascending order to obtain the ordered sequence W’, and the position index sequence *WT* = {*wt* (1), *wt* (2), *wt* (3), …, *wt* (8)} of each element in W. For example, if *W* = {5, 6, 8, 9, 7, 2, 3, 4}, we can obtain W’ = {2, 3, 4, 5, 6, 7, 8, 9} and the corresponding *WT* = {6, 7, 8, 1, 2, 5, 3, 4}.

**Step 3:** Convert the pixel value *P*’(*i*) in the scrambled image P’ into a binary form to generate an array *P*_Bit_ = {*Bit* (1), *Bit* (2), *Bit* (3), …, *Bit* (8)}. For example, if *P’* (*i*) = 176, the corresponding *P_Bit_* = {1, 0, 1, 1, 0, 0, 0, 0}. Use *WT* to scramble *P_Bit_* = {*Bit* (1), *Bit* (2), *Bit* (3), …, *Bit* (8)}, and a new form of reordering pixel value bits after scrambling is obtained. The scrambling method is similar to the formula (4). For example, if *P_Bit_* = {1, 0, 1, 1, 0, 0, 0, 0}, *WT* = {6, 7, 8, 1, 2, 5, 3, 4}, then the binary digit arrangement form of the point after bit scrambling will change to *P*’*_Bit_* = {0, 0, 0, 1, 0, 1}. Convert the scrambled binary number *P*’*_Bit_* to a decimal number to obtain the *i*th intermediate ciphertext pixel value *C’*(*i*) = 19.

Perform the step 2–step 3 operation until all the pixel values of the scrambled image *P*’ have obtained the intermediate ciphertext image sequence *C*’ = {*c*’(1), *c’*(2), …, *c’*(*mn*)}.

**Step 4:** Use the following Equations (5) and (6) to re-encrypt the intermediate ciphertext *C*’ to obtain the final ciphertext sequence *C* = {*c*(1), *c*(2), …, *c*(*mn*)}.

(5) $$s(i)=\mathrm{mod}(d\left(i\right)\times 2^{48},256)$$

(6) $$c(i)=\mathrm{mod}(s(i)+c^{\prime }(i),256)⊕c(i-1),i=1,2,\ldots ,mn$$

In particular, *c*(0) is required for the encryption of the first point (*i* = 1), and *c*(0) = 98 is a constant. The decryption process is the reverse process of the encryption process, which is not repeated here.

In this stage, we especially noticed that when generating the chaotic sequence D, the parameter *a*_2_ and the iteration number *K*_2_ of the Tent map were not correlated with the plaintext image. That is to say, the generation of the chaotic sequence D was independent of the plaintext image, which is the key to cracking the encryption algorithm. It can be seen that the operation in this stage completely depended on the position index sequence *WT* and the sequence *S* = (*s*(1), *s*(2), …, *s*(*mn*)), and the generations of these two sequences are completely transformed from the chaotic sequence *D*, while the generation of *D* has no relationship with the image to be encrypted.

## 3. Security Analysis of Original Image Chaotic Encryption Algorithm

From the encryption process of the original algorithm, we can see that the equivalent keys of the entire encryption algorithm are the scrambling sequence *T*, the pixel bit position scrambling sequence *WT,* and the diffusion sequence *S*. The generation of the scrambling sequence *T* is related to the sum of all the pixel values of the plaintext image, while the generations of *WT* and *S* are transformed from the chaotic sequence *D*, and the generation of *D* is not correlated with the image to be encrypted. That is to say, *WT* and *S,* which are used to encrypt different plaintext images, remain unchanged, which is the key to cracking the original algorithm. Next, we will crack the sequence *WT* and *S* one by one using the chosen plaintext attack method. Then, the global scrambling sequence *T* is cracked based on the assumption that the sum of all pixels of the plaintext image is an input parameter. The so-called chosen plaintext attack [27] means that the attacker temporarily obtains the right to use the encryption machine. Therefore, he can encrypt any plaintext and obtain the corresponding ciphertext to decode all or part of the plaintext and keys.

### 3.1. Cracking Diffusion Sequence S

The specific steps to crack sequence S are as follows:

**Step 1:** Use the original algorithm to encrypt an image *P*_0_ with the same size as the target ciphertext and all zero pixel values. It can be seen from the encryption process that pixel scrambling and bit position scrambling have no effect on *P*_0_; that is, the intermediate ciphertext *C*_0_ obtained after pixel scrambling and the bit position scrambling satisfies *C*_0_ = *P*_0_. Therefore, the ciphertext C obtained by encrypting the image *P*_0_ is shown in the Formula (7):

(7) $$\;\left\{\begin{array}{l} c(1)=s(1)⊕c(0)\\ c(i)=s(i)⊕c(i-1) \end{array}\right.$$

**Step 2:** S can be solved from Equation (7), as shown in the Formula (8).

(8) $$\left\{\begin{array}{l} s(1)=c(1)⊕c(0),i=1\\ s(i)=c(i-1)⊕c(i),i\neq 1 \end{array}\right.$$

### 3.2. Cracking Equivalent Bit Scrambling Matrix WT

In order to obtain the equivalent position matrix, the *WT* of bit scrambling, eight images *TP_i_*, and a pixel value of $2^{i-1}$ are constructed, *i* ∈ [1, 2, 3, 4, 5, 6, 7, 8]

$$TP_{1}={\left[\begin{array}{cccc} 2^{0} & 2^{0} & \ldots & 2^{0}\\ 2^{0} & 2^{0} & \ldots & 2^{0}\\ \ldots & \ldots & \ldots & \ldots \\ 2^{0} & 2^{0} & \ldots & 2^{0} \end{array}\right]}_{m\times n}\ldots \ldots TP_{8}=\left[\begin{array}{cccc} 2^{7} & 2^{7} & \ldots & 2^{7}\\ 2^{7} & 2^{7} & \ldots & 2^{7}\\ \ldots & \ldots & \ldots & \ldots \\ 2^{7} & 2^{7} & \ldots & 2^{7} \end{array}\right]$$

Input the constructed eight images *TP_i_* into the original encryption algorithm to obtain the corresponding ciphertexts *TC_i_*, and then use the equivalent key *S* decoded in step 1 to obtain eight intermediate ciphertexts *TC_i_^’^*. Taking *TC*_1_’ as an example, suppose that *TC*_1_(1, 1) = 64, and it is converted into the following binary (0, 1, 0, 0, 0, 0, 0, 0, 0), it can be found that the pixel value changes from the initial (0, 0, 0, 0, 0, 0, 0, 1) to (0, 1, 0, 0, 0, 0) after bit scrambling; that is, the bit scrambling encryption process change the digit 1 from the eighth-bit before encryption to the second bit after encryption, compared to the scrambling rule of the *WT*(1, 1) position that can be obtained, The rule changes of other positions can also be obtained. The changes are stored in the matrix *WT*1, which is the equivalent scrambling rule matrix of the first bit of the eight bit binary number with bit scrambling. The same method can obtain all 8-bit digital bit scrambling rules and store the obtained transformation rules in the matrix *WT* with size *m × n × 8. WT* is the complete equivalent key of bit scrambling.

### 3.3. Cracking of Global Scrambling Sequence T of Pixel Position

For the ciphertext image C to be cracked, the scrambled image *P*’ can be recovered according to the equivalent key streams *WT* and *S,* which were cracked in the previous Section 3.1 and Section 3.2. Since the scrambling operation does not change the pixel value of the image, the sum of the pixel values of plaintext image *P* can be calculated from *P*’. Our cracking algorithm is based on the assumption that the sum of all pixels in the plaintext image is an input parameter.

If the sequence *P*’ is converted into a matrix *A*’ of *m* × *n* in Section 2.2.1, the scrambling process in Section 2.2.1 is equivalent to the following scrambling process:

*A*’(*i*’, *j*’) = *A*(*i*, *j*)

That is, the pixel value of (*i*, *j*) is assigned the position of the original image matrix *A* to the pixel value of (*i*’, *j*’) of *A*’.The correspondence between position (*i*, *j*) and position (*i*’, *j*’) depends on an *m* × *n* matrix *AT*, and *AT* depends entirely on the scrambling sequence *T*. For example, for a 3 × 4 matrix *AT.*

$$AT=\left[\begin{array}{cccc} \left(2,1\right) & \left(3,4\right) & \left(1,1\right) & \left(3,2\right)\\ \left(3,1\right) & \left(1,4\right) & \left(2,2\right) & \left(3,3\right)\\ \left(1,3\right) & \left(2,4\right) & \left(1,2\right) & \left(2,3\right) \end{array}\right]$$

According to matrix *AT*, the pixel value of (2, 1) is assigned the position of the original image matrix *A* to the pixel value of (1, 1) for the position of *A*’, the pixel value of (3, 4) is assigned the position of *A* to the pixel value of the (1, 2) position of *A*’, the pixel value of the (1, 1) position of *A* is assigned to the pixel value of the (1, 3) position of *A*’, the pixel value of the (2, 3) position is assigned to the pixel value of (3, 4) position of *A*’, and so on. Therefore, cracking the scrambling sequence *T* is equivalent to cracking the matrix *AT*. Here, we break the matrix *AT* into two cases.

#### 3.3.1. The Situation That the Number of Lines and Columns of Ciphertext Image C to Be Cracked Does Not Exceed 256

In this case, only two special plaintext images needed to be constructed. The construction matrix *P*_1_ reflects the change rule of each column, and *P*_2_ reflects the change rule of each row.

$$P_{1}={\left(\begin{array}{cccc} 1 & 2 & \ldots & n\\ 1 & 2 & \ldots & n\\ \ldots & \ldots & \ldots & \ldots \\ 1 & 2 & \ldots & n \end{array}\right)}_{m\times n}P_{2}={\left(\begin{array}{cccc} 1 & 1 & 1 & 1\\ 2 & 2 & 2 & 2\\ \ldots & \ldots & \ldots & \ldots \\ m & m & m & m \end{array}\right)}_{m\times n}$$

Here, *m* < 256, *n* < 256.

Input *P*_1_ and *P*_2_ into the encryption system to obtain the corresponding ciphertext *C*_1_ and *C*_2_, and use the equivalent keys *S* and *WT,* recovered from 3.1 and 3.2 to recover the ciphertext *P*_1_′ and *P*_2_′ after the global scrambling of the pixel values. The equivalent global scrambling matrix *AT* can be obtained by *P*_1_′ and *P*_2_′. For example:

$$P_{1}=\left(\begin{array}{cccc} 1 & 2 & 3 & 4\\ 1 & 2 & 3 & 4\\ 1 & 2 & 3 & 4 \end{array}\right)\;P_{2}=\left(\begin{array}{cccc} 1 & 1 & 1 & 1\\ 2 & 2 & 2 & 2\\ 3 & 3 & 3 & 3 \end{array}\right)$$

according to

$$AT=\left[\begin{array}{cccc} \left(2,1\right) & \left(3,4\right) & \left(1,1\right) & \left(3,2\right)\\ \left(3,1\right) & \left(1,4\right) & \left(2,2\right) & \left(3,3\right)\\ \left(1,3\right) & \left(2,4\right) & \left(1,2\right) & \left(2,3\right) \end{array}\right]$$

Scramble *P*_1_ and *P*_2_ to obtain P1′ and P2′, respectively.

$$P_{1}^{\prime }=\left(\begin{array}{cccc} 1 & 4 & 1 & 2\\ 1 & 4 & 2 & 3\\ 3 & 4 & 2 & 3 \end{array}\right),P_{2}^{\prime }=\left(\begin{array}{cccc} 2 & 3 & 1 & 3\\ 3 & 1 & 2 & 3\\ 1 & 2 & 1 & 2 \end{array}\right)$$

*P*_1_′ determines the column label, and *P*_2_′ determines the row label. Therefore, the matrix determined by *P*_1_′and *P*_2_′ is *AT*’ is equal to *AT.*

$$AT^{\prime }=\left[\begin{array}{cccc} \left(2,1\right) & \left(3,4\right) & \left(1,1\right) & \left(3,2\right)\\ \left(3,1\right) & \left(1,4\right) & \left(2,2\right) & \left(3,3\right)\\ \left(1,3\right) & \left(2,4\right) & \left(1,2\right) & \left(2,3\right) \end{array}\right]$$

#### 3.3.2. The Situation That the Number of Lines and Columns of Cracked Ciphertext Image C Is Greater than 256

In this case, we have the following cracking steps.

**Step 1:***L*_c_ special plaintext images I*_ck_* (*k* = 0, 1, …, L_c_−1) are required to construct a matrix reflecting the change rule of each column, and the size of each *I_ck_* is *m* × *n*. The element values of column *i* of matrix *I_ck_* are the same and satisfy Formula (10). Moreover, *L_c_* meets Formula (9) [28]

(9) $$L_{c}=ceil(\log_{256}n)$$

(10) $$I_{ck}(i)=floor(i\times 256^{-k})\;\mathrm{mod}\;256,\;i=1,2,3,\ldots ,n.$$

where the ceil (*x*) means rounding up the *x* and the floor (*x*) means rounding down *x*.

To explain the problem more clearly, suppose *m* = 294 and *n* = 289. *L_c_* = 2 special plaintext images *I_c_*_0_ and *I_c_*_1_ need to be constructed according to Equation (9), and *I_c_*_0_ and *I_c_*_1,_ which were constructed according to Equation (10), have the following forms:

$$I_{c0}={\left(\begin{array}{ccccccccc} 1 & 2 & \ldots & 255 & 0 & 1 & 2 & \ldots & 33\\ 1 & 2 & \ldots & 255 & 0 & 1 & 2 & \ldots & 33\\ 1 & 2 & \ldots & 255 & 0 & 1 & 2 & \ldots & 33\\ 1 & 2 & \ldots & 255 & 0 & 1 & 2 & \ldots & 33\\ 1 & 2 & \ldots & 255 & 0 & 1 & 2 & \ldots & 33\\ 1 & 2 & \ldots & 255 & 0 & 1 & 2 & \ldots & 33\\ \ldots & \ldots & \ldots & \ldots & \ldots & \ldots & \ldots & \ldots & \ldots \\ 1 & 2 & \ldots & 255 & 0 & 1 & 2 & \ldots & 33\\ 1 & 2 & \ldots & 255 & 0 & 1 & 2 & \ldots & 33 \end{array}\right)}_{294\times 289}I_{c1}={\left(\begin{array}{ccccccccc} 0 & 0 & \ldots & 0 & 1 & 1 & 1 & \ldots & 1\\ 0 & 0 & \ldots & 0 & 1 & 1 & 1 & \ldots & 1\\ 0 & 0 & \ldots & 0 & 1 & 1 & 1 & \ldots & 1\\ 0 & 0 & \ldots & 0 & 1 & 1 & 1 & \ldots & 1\\ 0 & 0 & \ldots & 0 & 1 & 1 & 1 & \ldots & 1\\ 0 & 0 & \ldots & 0 & 1 & 1 & 1 & \ldots & 1\\ \ldots & \ldots & \ldots & \ldots & \ldots & \ldots & \ldots & \ldots & \ldots \\ 0 & 0 & \ldots & 0 & 1 & 1 & 1 & \ldots & 1\\ 0 & 0 & \ldots & \;\;\;0_{col=255} & 1 & 1 & 1 & \ldots & 1 \end{array}\right)}_{294\times 289}$$

Input *I_c_*_0_ and *I_c_*_1_ into the original encryption system to obtain the corresponding ciphertext *C*_0_ and *C*_1_. Use the equivalent keys obtained in Section 3.1 and Section 3.2 to recover the ciphertext *I_c_*_0_’ and *I_c_*_1_′ of *I_c_*_0_ and *I_c_*_1_ after the global scrambling of pixel values. Then, according to Formula (11), use *I_c_*_0_′ and *I_c_*_1_′ to construct matrix *P*_1_′, which reflects the change rule of each column.

(11) $$P_{1}^{\prime }=\sum_{k=0}^{L_{c}-1}I^{\prime }{}_{ck}\times 256^{k}=\sum_{k=0}^{1}I^{\prime }{}_{ck}\times 256^{k}$$

**Step 2:***L_r_* special plaintexts *I_rk_* (*k* = 0,1,…, *L_r_*−1) are required to construct a matrix reflecting the change rule of each row and the size of *I_rk_* is *m* × *n*. The element values of row *i* of matrix *I_rk_* are the same and satisfy formula (13). Moreover, *L_r_* meets the Formula (12) [28]

(12) $$L_{r}=ceil(\log_{256}m)$$

(13) $$I_{rk}(i)=floor(i÷256^{k})\%256,i=1,2,3,\ldots ,\;m$$

where ceil (*x*) means rounding up the *x* and floor (*x*) means rounding down *x*.

To explain this problem more clearly, suppose *m* = 294 and *n* = 289. Then, *L_r_* = 2 special plaintext images *I_r_*_0_ and *I_r_*_1,_ need to be constructed according to Equation (12). *I_r_*_0_ and *I_r_*_1,_ constructed according to Equation (13), have the following forms:

$$I_{r0}={\left(\begin{array}{ccccc} 1 & 1 & 1 & \ldots & 1\\ 2 & 2 & 2 & \ldots & 2\\ 3 & 3 & 3 & \ldots & 3\\ \ldots & \ldots & \ldots & \ldots & \ldots \\ 255 & 255 & 255 & \ldots & 255\\ 0 & 0 & 0 & \ldots & 0\\ 1 & 1 & 1 & \ldots & 1\\ 2 & 2 & 2 & \ldots & 2\\ \ldots & \ldots & \ldots & \ldots & \ldots \\ 39 & 39 & 39 & \ldots & 39 \end{array}\right)}_{294\times 289}I_{r1}={\left(\begin{array}{ccccc} 0 & 0 & 0 & \ldots & 0\\ 0 & 0 & 0 & \ldots & 0\\ 0 & 0 & 0 & \ldots & 0\\ \ldots & \ldots & \ldots & \ldots & \ldots \\ 0 & 0 & 0 & \ldots & 0_{row=255}\\ 1 & 1 & 1 & \ldots & 1\\ 1 & 1 & 1 & \ldots & 1\\ 1 & 1 & 1 & \ldots & 1\\ \ldots & \ldots & \ldots & \ldots & \ldots \\ 1 & 1 & 1 & \ldots & 1 \end{array}\right)}_{294\times 289}$$

Input *I_r_*_0_ and *I_r_*_1_ into the original encryption system to obtain the corresponding ciphertext *C*_0_ and *C*_1_ and recover *I_r_*_0_′ and *I_r_*_1_′ of *I_r_*_0_ and *I_r_*_1_ only after the global scrambling of pixel values by using the equivalent keys obtained in Section 3.1 and Section 3.2. According to Formula (14), use *I_r_*_0_′ and *I_r_*_1_′ to construct matrix *P*_2_′, which reflects the change rule of each row:

(14) $$P_{2}^{\prime }=\sum_{k=0}^{L_{r}-1}I^{\prime }{}_{rk}\times 256^{k}=\sum_{k=0}^{1}I^{\prime }{}_{rk}\times 256^{k}$$

Matrix *P*_1_′ determines column labels, and matrix *P*_2_′ determines row labels. The equivalent global scrambling matrix *AT* can be obtained through the matrix *P*_1_′ and the matrix *P*_2_′. So far, all the equivalent key streams of the original algorithm were cracked by 9 + ceil(log_256_*m*) + ceil(log_256_*n*) special plaintext images.

## 4. Experimental Simulation

**Figure 2:**
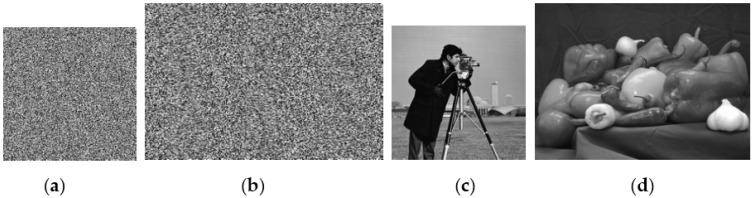
Simulation results of chosen plaintext attack. (**a**) The 256 × 256 ciphertext to be decoded; (**b**) The 384 × 512 ciphertext to be decoded; (**c**) The corresponding decoding result of (**a**); (**d**) The corresponding decoding result of (**b**).

In order to verify the effectiveness of our attack algorithm, an experimental simulation of chosen plaintext attacks was carried out according to the analysis in Section 3. The simulation experiment adopted the Matlab2015a platform and the images “cameraman” of size 256 × 256 and “pepper” of size 384 × 512, respectively. According to the previous analysis, eleven special plaintext images were required to crack the ciphertext image of size 256 × 256: a plaintext image with all pixel values of zero, eight plaintext images *TP_i_* with all pixel values of 2*^i^* ^− 1^, *i* ∈ [1, 2, 3, 4, 5, 6, 7, 8], and two plaintext images with pixel values such as image matrix *P*_1_ and *P*_2_ constructed in Section 3.3. Compared with cracking the ciphertext image of size 256 × 256, thirteen special plaintext images were required to crack the ciphertext image of size 384 × 512. A plaintext image with all pixel values of zero eight plaintext images *TP_i_* with all pixel values of 2*^i −^* ^1^, *i* ∈ [1, 2, 3, 4, 5, 6, 7, 8], and four plaintext images with pixel values such as image matrix *I_c_*_0_, *I_c_*_1_ and *I_r_*_0_, *I_r_*_1_ were constructed in Section 3.3. The simulation results are shown in Figure 2.

## 5. The Improved Algorithm and Its Decryption Algorithm

### 5.1. The Improved Encryption Algorithm

To sum up, the original image encryption algorithm had the following defects:

(1) The equivalent keys of the whole encryption algorithm are the scrambling sequence *T*, the pixel bit position scrambling sequence *WT,* and the diffusion sequence *S.* The generation of the scrambling sequence *T* is related to the sum of all pixel values of the plaintext image, while the generations *WT* and *S* are all the transformation of the chaotic sequence *D*, and the generation of *D* was not correlated with the image to be encrypted. That is to say, *WT* and *S* were used to encrypt different plaintext images and remained unchanged, which is the key to cracking the original algorithm.

(2) The operation in the bit scrambling stage and the operation in the diffusion stage are all too simple.

**Figure 3:**
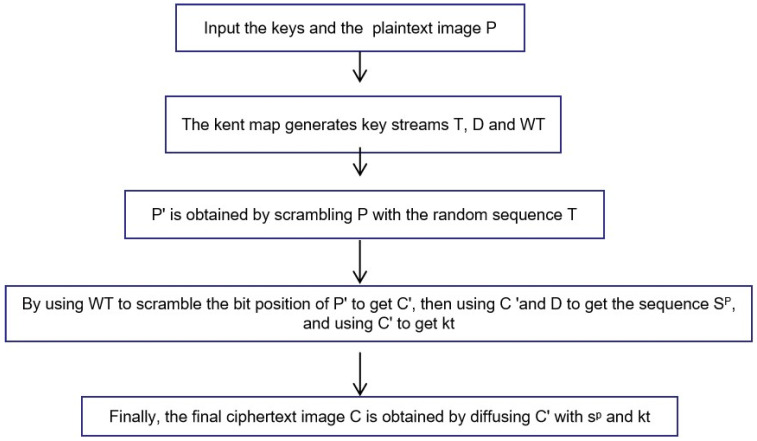
The flow chart of the improved encryption process.

In order to eliminate the safety defects of the original algorithm, we put forward some improvement measures on the basis of being loyal to the original algorithm. The algorithm flow chart of the improved algorithm is shown in Figure 3.

The steps of the improved encryption algorithm are as follows.

**Step 1:** Assume that the plaintext image matrix is A with size *m* × *n.* Scramble the plaintext image according to the method introduced in Section 2.2.1 to obtain the scrambled image sequence *P*’ = (*p*’(1), *p*’(2), …, *p*’(*mn*)).

**Step 2:** The bit-level scrambling operation is performed on *P*’ to obtain the middle ciphertext *C*’. The scrambling method is the same as the method in Section 2.2.2.

**Step 3:** Diffuse the intermediate ciphertext *C*’, which is greatly improved compared with the original algorithm, as follows:

(1) Use the middle ciphertext C’ to construct sequence *V^P^* = {*v^p^*(1), *v^p^*(2), *v^p^*(3), …, *v^p^*(*mn*)} according to Formula (15):

(15) $$v^{p}\left(i\right)=\left\{\begin{array}{l} \sum_{j=2}^{mn}c^{\prime }\left(j\right),if\;i=1\\ v^{p}\left(i-1\right)-c^{\prime }\left(i\right),if\;i=2,3,\ldots ,mn \end{array}\right.$$

(2) According to Equation (16), the sequence *S^P^* = {s*^p^*(1), s*^p^*(2), s*^p^*(3), …, s*^p^*(*mn*)} is obtained using the sequence *D* generated in the original algorithm, and the sequence *V^P^*.

(16) $$s^{p}\left(i\right)=\mathrm{mod}\left(\mathrm{floor}\left(\frac{v^{p}\left(i\right)\times d(i)}{256^{5}}\times 10^{12}\right),256\right)$$

(3) Generate sequence *kt* (*i*) by using intermediate ciphertext *C*’ = (*c*’(1), *c*’(2), …, *c*’(*mn*)) according to Formula (17):

(17) $$kt\left(i\right)=floor\left(c^{\prime }\left(i+1\right)\times \left(i-1\right)/256\right)+1,\;i=2,\;\ldots ,\;\mathrm{mn}-1.\;$$

which is obviously, *kt*(*i*) ∈ [1, *i −* 1].

(4) According to the Formula (18), the final cryptogram C is obtained by using the sequence *S^P^* = {*s^p^* (1), *s^p^*(2), *s^p^*(3), …, *s^p^*(*mn*)} and *kt*(*i*)

(18) $$\left\{\begin{array}{l} c(1)=\mathrm{mod}(s^{p}(1)+c^{\prime }(1),256)\\ c(i)=\mathrm{mod}(s^{p}(i)+c^{\prime }(i),256)⊕c(kt(i)),i=2,\ldots ,mn-1\\ c\left(mn\right)=\mathrm{mod}(s^{p}(mn)+c^{\prime }(mn),256) \end{array}\right.$$

The pseudocode of the improved encryption algorithm is shown in Algorithm 1.

**Algorithm 1:** The improved encryption algorithmInput: Plaintext image A and encryption keys. Output: Ciphertext image *C* Step 1: Generate key streams *t*(*i*), d(i), and *WT*(*i*) Step 2: Convert the digital image matrix A into a one-dimensional sequence *P* and scramble the plaintext sequence *P*. for i = 1:m*n  P’(*i*) = *P*(*t*(*i*)) End Step 3: Convert P’(*i*) into binary P_Bit_ (*i*), and then use *WT*(*i*) to scramble P_Bit_(*i*) to obtain P’_Bit_ (*i*) and convert P’_Bit_(*i*) into a decimal number C’(*i*) Step 4: Generate V^p^(i), S^p^(*i*) and Kt(*i*) V^p^(1) = sum(C’)−C’(1) S^p^(1) = mod(floor(V^p^(1)*d(1)*10^12^/256^5^),256) For i = 2:m*n  V^p^(i) = V^p^(i − 1)−C’(i − 1)  S^p^(i) = mod(floor(V^p^(i)*d(i)*10^12^/256^5^),256)  Kt(i) = floor(C’(i + 1)*(i − 1)/256) + 1 end Step 5: Generate the final ciphertext image. C(1) = mod(S^p^(1) + C’(1),256) For i = 2:m*n − 1 C(i) = mod(S^p^(i) + C’(i),256) ⊕ c(Kt(i)) End C(m*n) = mod(S^p^(m*n) + C’(m*n),256)

### 5.2. Decryption Algorithm of Improved Algorithm

The decryption process is the reverse of the encryption process. The specific steps are as follows:

**Step 1:** Set the control parameters of the chaotic system using the existing key, and iterate the Kent chaotic map to obtain the sequence *D* = (*d*(1), *d*(2), …, *d*(*mn*)) with the length of *mn*. From Equation (15), we can infer that *v^p^*(*mn*) = 0, and further infer that *s^p^*(*mn*) = 0 from Equation (16) and c’(*mn*) = c (*mn*) from equation (18), and further from Equation (15), we can infer that *v^p^*(*mn* − 1) = c’(*mn*) and obtain the value of *s^p^*(*mn* − 1) from the Equation (16) and at the same time, *kt*(*mn*) can be solved from the Equation (17). Finally, by using *s^p^*(*mn* − 1) and *kt*(*mn*), according to Equation (18), c’ (*mn* − 1) can be solved. By analogy, *c*’(*mn* − 2), *c*’(*mn* − 3), …, *c*’(2) are decrypted, respectively, and then *v^p^*(1) is calculated, and *c*’(1) is decrypted.

**Step 2:** Use the existing key, the C’ recovered in the previous step can be used to recover P’; the plaintext image matrix recovers A from P’. This process is the reverse process of the encryption process, which is not repeated here.

## 6. Experimental Simulation and Security Analysis of Improved Algorithm

### 6.1. Experimental Simulation

**Figure 4:**
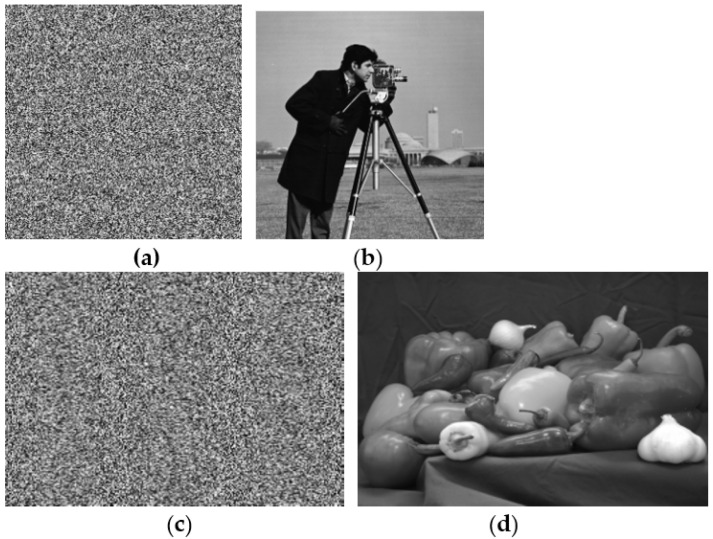
The encryption and decryption effect. (**a**) The 256 × 256 encrypted image. (**b**) The decrypt image of (**a**). (**c**) The 384 × 512 encrypted image. (**d**) The decrypted image of (**c**).

The key to the improved encryption algorithm is the same as that of the original algorithm. Set the initial value of tent mapping *x*_0_ = 0.3987623, *a*_2_ = 0.8739, *k*_2_ = 3000, the simulation experiment adopts matlab2015a platform, and the image “cameraman” of size 256 × 256 and the image “pepper” of size 384 × 512 are selected, respectively. The experimental results are shown in Figure 4. The experimental results show that the improved encryption algorithm has a good encryption effect and can decrypt without error.

### 6.2. Security Analysis of the Improved Algorithm

The improved algorithm is as faithful as possible to the original algorithm, including pixel position scrambling, bit-level scrambling, and pixel diffusion operations. Therefore, the improved algorithm has the same excellent characteristics as the original algorithm, such as good statistical characteristics, and the algorithm is sensitive to the plaintext or the keys. More importantly, the improved algorithm can resist the chosen plaintext attack.

#### 6.2.1. Information Entropy Analysis of Ciphertext Image

The more chaotic the ciphertext image is, the greater the image information entropy, the less information the ciphertext image provides, and the better the encryption effect. The calculation formula of information entropy is as follows:

(19) $$E=-\sum_{i=1}^{n}p_{i}\log_{2}(p_{i})$$

where *p_i_* is the probability of the occurrence of the *i*th order gray value. For 256-level grayscale images, *n* = 256. When the probability distribution of the ciphertext is equal to the probability distribution: that is, when the probability of each value between [0 255] is 1/256, the entropy is 8-bit, which is the maximum value.

The information entropy of the four digital images “rice”, “cameraman”, “autumn”, and “pepper” encrypted by the improved algorithm and the original algorithm is shown in Table 1. It can be seen that the information entropy of the four ciphertext images obtained by the improved algorithm is slightly larger than that obtained by the original algorithm, which is very close to 8 bits. It shows that the randomness and unpredictability of the encrypted image are very high.

#### 6.2.2. Pixel Correlation Analysis

For a natural image, adjacent pixels are very close, with strong correlation and great redundancy of image information. One of the goals of image encryption is to eliminate this redundancy and reduce the correlation between adjacent pixels. In order to evaluate the plaintext image and ciphertext image, we randomly selected 4000-pixel points as reference points, took the adjacent pixel points along the horizontal, vertical, and diagonal directions to form pixel pairs, and used the correlation coefficient formula (20) to calculate the correlation coefficient values of plaintext image and corresponding ciphertext image in these three directions, The correlation coefficients of adjacent elements of the obtained original image and ciphertext image are shown in Table 2.

(20) $$xc=\frac{n\sum_{i=1}^{n}x_{i}y_{i}-\sum_{i=1}^{n}x_{i}\sum_{i=1}^{n}y_{i}}{\sqrt{n\sum_{i=1}^{n}x_{i}^{2}-{\left(\sum_{i=1}^{n}x_{i}\right)}^{2}}\;\sqrt{n\sum_{i=1}^{n}y_{i}^{2}-{\left(\sum_{i=1}^{n}y_{i}\right)}^{2}}}$$

where *x_i_* and *y_i_* represent the gray values of the adjacent two pixels, respectively, and *n* represents the number of selected pairs of pixels.

It can be seen from Table 2 that there are strong linear relationships between adjacent pixels of plaintext images in three directions, while the relationships between adjacent pixels of ciphertext images in three directions are random, which means that the redundancy and correlation of the pixels are eliminated.

#### 6.2.3. Analysis of the Algorithm Sensitivity to the Plaintext Image

The sensitivity of the encryption algorithm to the plaintext image means that the encrypted ciphertext image is completely different from the original ciphertext image, even if there is only a small change in the plaintext image. The sensitivity of encryption algorithms to plaintext can be measured by using the concept of the number of pixels change rate (NPCR) and unified average change intensity (UACI). The calculation formulas of NPCR and UACI are (21) and (22), respectively.

(21) $$NPCR=\frac{\sum_{i=1}^{M}\sum_{j=1}^{N}D_{ij}}{M\times N}\times 100\%$$

(22) $$UACI=\frac{\left(\sum_{i=1}^{M}\sum_{j=1}^{N}\left(\frac{\left|c(i,j)-c^{\prime }(i,j)\right|}{255}\right)\right)}{M\times N}\times 100\%$$

where:

(23) $$D_{ij}=\left\{\begin{array}{c} 1,c(i,j)\neq c^{\prime }(i,j)\\ 0,c(i,j)=c^{\prime }(i,j) \end{array}\right.$$

where *M* × *N* is the size of the image, *c*(*i*, *j*) represents the pixel in a coordinate (*i*, *j*) of the ciphertext image corresponding to the original plaintext image, and *c*’(*i*, *j*) represent the pixel in a coordinate (*i*, *j*) of the ciphertext image corresponding to the changed plaintext image. For 256-bit grayscale images, the expected values of NPCR and UACI are 99.6094% and 33.4635%, respectively. In the improved algorithm, 200 pixels in each image were randomly selected, and their pixel values were changed. The maximum, minimum, average and maximum, minimum, and average values of NPCR and UACI calculated from the results are listed in Table 3, respectively. At the same time, the original algorithm was used for the same operation. The maximum, minimum, average and maximum, minimum, and average values of NPCR and UACI are listed in Table 4, respectively. They are very close to ideal values. It can be seen that the change in the gray value of a pixel in the original image led to the change in almost all the gray values of pixels in the improved encrypted image and the original encryption algorithm.

#### 6.2.4. Key Sensitivity Analysis

**Figure 5:**
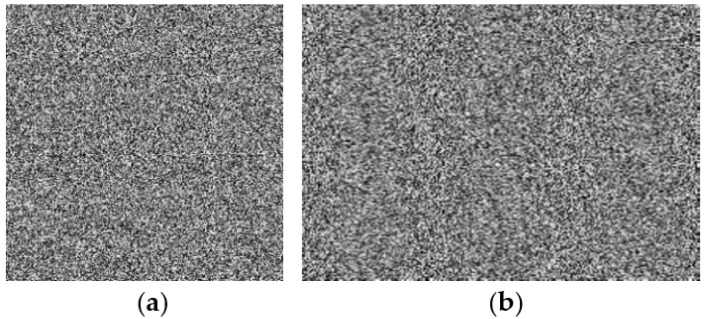
The decrypted images by using the error keys. (**a**) Decryption result of “cameraman”. (**b**) Decryption result of “peppers”.

A secure encryption algorithm should be sensitive to the key in order to resist brute-force attacks. Key sensitivity means that if the decryption key is slightly different from the correct key, no useful information about the plaintext image can be obtained from the decryption result. We set the initial value of tent mapping *x*_0_ = 0.3987623 + 10^−10^, *a*_2_ = 0.8739, *k*_2_ = 3000 to decrypt the original ciphertext images of Figure 4a,c, and the decryption result is shown in Figure 5. It can be seen that no information about the original image can be obtained in the decrypted image, which also shows the high sensitivity of the algorithm to the key. In the same way, if the keys *a*_2_ and *K*_2_ have a slight error, the decrypted image will also be a chaotic image.

#### 6.2.5. Analysis of Anti Chosen-Plaintext Attack

The ability of the improved algorithm to resist the chosen-plaintext attack is emphatically analyzed. Obviously, compared with the original algorithm, it can be seen that the random key streams *s*^p^(*i*) and *kt*(*i*) used in the diffusion phase were related to the intermediate ciphertext *C*’ from Formulas (15)–(17). That is, the *s^p^*(*i*) and *kt*(*i*) used to encrypt different images was different. Therefore, the *s^p^*(*i*) and *kt*(*i*) obtained by the chosen-plaintext attack were different from the *s^p^*(*i*) and *kt*(*i*) used in the cracked target image. So the algorithm can resist the chosen-plaintext attack. On the other hand, the decryption key of the improved algorithm was the same as that of the original algorithm. The improved algorithm can resist the attack of the chosen plaintext and has the encryption effect of a “one time pad” but does not increase the burden of key transmission.

#### 6.2.6. Time Cost Analysis

The time cost is an important index for evaluating encryption algorithms. The time cost and test results of several images are shown in Table 5 below.

The time cost of the encryption process mainly includes the generation of chaotic sequencing, the scrambling of the spatial domain, the scrambling of bit, and the diffusion operation. The time cost of the decryption process mainly includes the generation of chaotic sequence, the space domain inversion scrambling, the bit inversion scrambling operation, and the diffusion operation. Therefore, we can see that the proposed algorithm can show fast speeds.

## 7. Conclusions

This paper analyzes the security of an image encryption algorithm with the double scrambling of the pixel position and bit and finds its security loopholes. According to the method of chosen-plaintext attack, for a ciphertext grayscale image with a size of *m* × *n*, only 9 + ceil(log_256_*m*) + ceil(log_256_*n*) special plaintext grayscale images and their corresponding ciphertext images are required to obtain the equivalent keys, thus realizing the cracking of the original chaotic encryption algorithm. A simple numerical example and several simulation experiments demonstrate the effectiveness of the proposed attack method. From the encryption process of the original algorithm, it can be seen that the equivalent keys of the entire encryption algorithm are the scrambling sequence *T*, the pixel bit position scrambling sequence *WT,* and the diffusion sequence *S*. The generation of the scrambling sequence *T* is related to the sum of all pixel values of the plaintext image, while the generations of *WT* and *S* are the transformation of the chaotic sequence *D*, and the generation of *D* is not correlated with the image to be encrypted. That is to say, *WT* and *S,* which are used to encrypt different plaintext images, remain unchanged, which is the key to cracking the original algorithm. In order to solve the security defects of the original algorithm, an improved algorithm has been proposed to overcome the defect, which can resist the chosen-plaintext attack and has the encryption effect of a “one time pad”. Finally, various security analyses are carried out for the improved algorithm.

## Figures and Tables

**Table 1:** Information entropy of encrypted image.

Images	The Original Algorithm	The Improved Algorithm
rice	7.9926	7.9984
cameraman	7.9893	7.9978
autumn	7.9897	7.9932
pepper	7.9943	7.9958

**Table 2:** The correlation coefficient comparison between original image and encrypted image.

Images	Horizontal	Vertical	Diagonal
The plaintext image of“Rice”	0.9667	0.9243	0.9099
The ciphertext image of“Rice”	−0.0012	0.0147	−0.0191
The plaintext image of“Cameraman”	0.9609	0.9508	0.9365
The ciphertext image “Cameraman”	−0.0030	−0.0169	0.0085
The plaintext image of“autumn”	0.9709	0.9887	0.9809
The ciphertext image “autumn”	−0.0068	0.0108	−0.0109
The plaintext image of“pepper”	0.9809	0.9798	0.9832
The ciphertext image of“pepper”	0.0043	0.0184	−0.0193

**Table 3:** NPCR and UACI test results for the improved encryption algorithm.

Images	NPCR%			UACI%		
	Max	Min	Average	Max	Min	Average
Rice	99.8634	99.7026	99.7662	33.5587	33.3998	33.4819
autumn	99.7932	99.6241	99.7310	33.6998	33.2584	33.4689
pepper	99.9532	99.4897	99.6859	33.7983	33.3619	33.5418
camera	99.7898	99.5698	99.6677	33.5492	33.2898	33.3598

**Table 4:** NPCR and UACI test results for the original encryption algorithm.

Images	NPCR%			UACI%		
	Max	Min	Average	Max	Min	Average
Rice	99.8543	99.6826	99.7062	33.4569	33.3916	33.4819
autumn	99.6687	99.5821	99.6315	33.6754	33.4508	33.6569
pepper	99.9438	99.5839	99.6656	33.8543	33.4619	33.6418
camera	99.7998	99.4668	99.5657	33.5487	33.2858	33.3578

**Table 5:** Test result of time cost (units).

Images	Size	The Encryption Time	The Decryption Time
Rice	256 × 256	0.034876	0.049487
autumn	206 × 345	0.094978	0.129496
peppers	384 × 512	0.110982	0.130679
camera	256 × 256	0.034567	0.051543

## Data Availability

Data sharing is not applicable.
